# Chondrosarcoma of the Proximal Humerus Secondary to Ollier Disease: An 8-Year Follow-Up of Successful Resection of the Tumor With Endoprosthetic Replacement of the Proximal Humerus

**DOI:** 10.14740/jocmr1777w

**Published:** 2014-03-31

**Authors:** Kang Zheng, Zhao Xiang Peng, Ping Pin Zheng

**Affiliations:** a Department of Orthopedics, Li hui li Hospital, Ningbo Medical Center, Ningbo, Zhejiang, China; b Department of Pathology, Erasmus Medical Center, Rotterdam, The Netherlands

**Keywords:** Ollier’s disease, Chondrosarcoma, Humeral neoplasms, Limb salvage, Prosthetic replacement, Reconstructive surgical procedures, Long-term follow-up

## Abstract

We present a 25-year-old male patient with a diagnosis of multiple enchondromatosis, who developed chondrosarcoma on the proximal humerus of the right upper limb. The patient had the pre-existing lesions of Ollier’s disease discovered during his childhood. The patient underwent wide resection of the sarcoma with a prosthetic replacement of the proximal humerus. So far we have followed up the patient for 8 years with no evidence of local recurrence and/or metastasis. The therapeutic results have been satisfied with a good functional recovery of the treated limb, enabling the patient to return to the pre-disease daily living and occupational activities. The reconstructive procedures represent an effective surgical strategy for limb salvage in the treatment of large segmental defects after resection of humeral tumors, substantially solving the functional and esthetic problems due to such a wide resection, and significantly improving the quality of life for the patient.

## Introduction

Ollier disease is a rare non-hereditary skeletal disorder characterized by the presence of multiple enchondromas (enchondromatosis), and these cartilaginous lesions can be limited to one limb, or localized to one half of the body [1, 2]. The disease often manifests in infancy and early childhood, usually starting with the appearance of palpable and painless bony masses on the involved limbs. The condition in which multiple enchondromatosis is associated with soft tissue hemangiomas is known as Maffucci syndrome [2]. With progressive skeletal growth, the lesions become more evident and characteristic, resulting in cosmetic problems and functional deformities, for example, deformity, limb asymmetry, pathologic fractures and potential risk for malignant change [2]. Malignant transformation of an enchondroma represents a well-known complication of Ollier’s disease [3-7]. Approximate 30-40% of the patients with multiple enchondromas will develop a malignant bone neoplasm, most probably chondrosarcoma [8-10]. There is no specific medical treatment for Ollier disease. Surgery is indicated in case of complications (pathologic fractures, growth defect and malignant transformation) [2]. More importantly, surgical strategies remain central to the management of those patients with malignant transformation because of the low efficacy of adjuvant chemotherapy and radiotherapy [11]. The prognosis for Ollier disease is difficult to predict, dependent on the nature of the complications and the severity of the involvement as well as the surgical management. It is still unclear whether the disorder is caused by a single gene defect or by combinations of (germ-line and/or somatic) mutations [12]. The diagnosis is mainly based on clinical and radiologic evaluations. Histologic analysis has a limited role and is mainly used if malignancy is suspected [2]. It should be aware that the radiologic manifestations must be taken into account during making a histopathologic diagnosis. Here we report a case with chondrosarcoma of the proximal humerus secondary to Ollier disease with treatment of wide resection of the tumor and a prosthetic replacement of the proximal humerus. So far an 8-year follow-up has been done for the patient, and the patient remains well with normal daily living and occupational activities.

## Case Report

**Figure 1:**
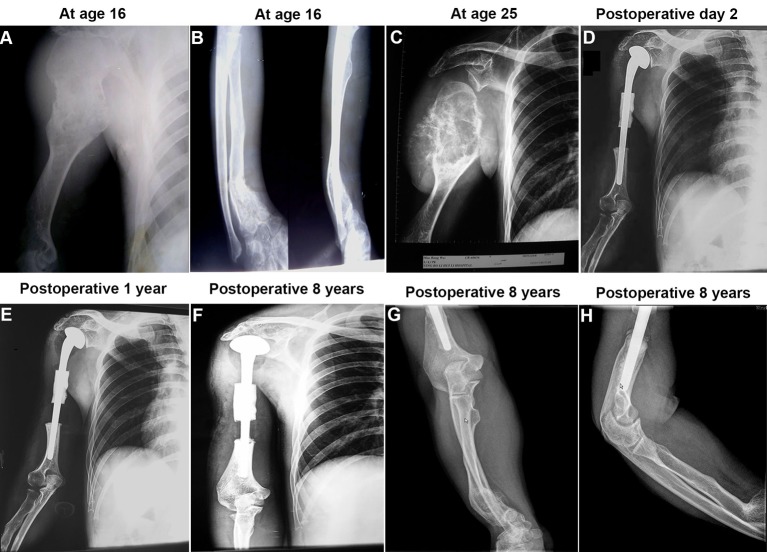
Radiographic evaluation of the surgical treatment at pre- and postoperative stages. (A and B) Radiograph showing cartilaginous lesions or enchondromas predominantly localized in the upper part of the humerus (A) and the lower part of the radius (B) of the 16 years old boy affected with Ollier disease. (C) A huge tumor of the proximal humerus with massive cortical destruction and soft tissue extension was noticed at age 25 of the patient. (D) The radiograph on the postoperative day 2 (anteroposterior view). The prosthesis was well in place after the surgery. (E) The radiograph on 1 year after the surgery (anteroposterior view). The prosthesis was still well in place. (F) The radiograph on 8 years after the surgery (anteroposterior view). The prosthesis remained well in place. (G and H) The radiographs of the ulna and radius of the limb were also taken at age 33 of the patient (8 years after the surgery). There were no signs for malignant transformation in those lesions.

A 25-year-old male patient was admitted to our department, presenting progressive pain and numbness with a rapidly growing mass located in the upper part of the right arm for 2 months in 2005. At the time of the hospital admission, he had completely lost his occupational capacity of the arm (100% unable to work). Physical examination revealed a palpable mass on the upper part of his right arm with a size 13 × 12 cm. Radiographic examination demonstrated a large mass over 10 cm in diameter on the proximal humerus, with massive cortical erosion, extension of the tumor into soft tissues and indistinctness of the surface of the tumor (Fig. 1C). A diagnosis of chondrosarcoma was made in view of the radiologic findings in combination with the histology by the needle biopsy. The specimen was histologically classified as grade II chondrosarcoma. The patient had a 20-year history of a painless and palpable bony mass on this location and had gradually increased in size. He first sought treatment at age 16 because he had a distal radius fracture. At that period of time, the plain radiographs demonstrated multiple cartilaginous dysplasia or multiple enchondromatosis (Ollier’s disease) in the humerus with most evidently involving the upper part of the humerus (Fig. 1A), and the lower part of the radius (Fig. 1B) of the right upper limb. Soft tissue of hemangiomas was not detected. The patient had no family history of skeletal disorders and no other medical history of note.

**Figure 2:**
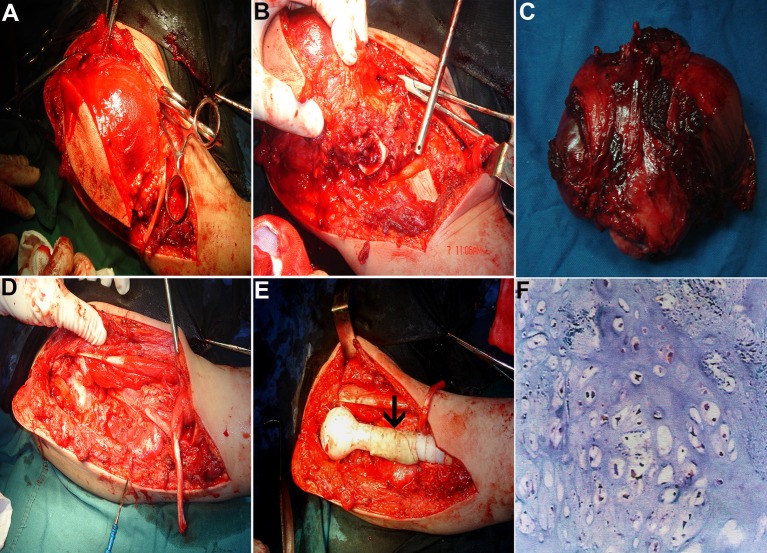
Wide resection of the tumor with a prosthetic replacement of the proximal humerus. (A) The range of tumor resection. (B) The humeral osteotomy was made 2 cm distal to the tumor extent to ensure a wide margin. (C) Overview of the resected tumor. (D) The tumor resection cavity. (E) Placement of the proximal humeral prosthesis. The shaft preparation with cementing the prosthesis (arrow indicated) was designed for supporting the reattachment of muscles and/or tendons to the prosthesis. (F) Histologic confirmation for the diagnosis of chondrosarcoma of the proximal humerus.

A limb-salvage strategy with treatment of the large segmental defects following resection of the tumor and a prosthetic replacement of the proximal humerus was designed for the patient. The informed consent was obtained from the patient prior to the operation. The tumor resection prosthesis was applied in the same session following the tumor resection (Fig. 2A-E). The tumor tissues were excised with maximum possible surgical safety margins. Frozen section biopsy was conducted during the surgery to ensure a safe surgical margin. Then the humeral head prosthesis (Waldemar LINK GmbH & Co., Germany) tailored for the patient was implanted, and the shaft preparation with cementing the prosthesis (Fig. 2E, arrow indicated) was designed for supporting the reattachment of muscles and/or tendons to the prosthesis. This strategy was designed to get better active function, because inadequate reattachment of muscles and/or tendons to prosthesis cause considerable loss of function and loss of joint stability [13, 14]. No adjuvant chemotherapy and radiotherapy for the patient were given. Postoperative physiotherapy was applied to this patient. After surgery, the patient was to start active motion of the hand, wrist and elbow at 1 day even with mild tolerable pain. The patient could start to swing the shoulder joint at 4 weeks after surgery. Lifting and abduction of the shoulder joint were gradually started at 6 weeks after surgery. Early full weight bearing was started at 8 weeks postoperatively. Three months after surgery, the patient was almost 100% back to work and could perform all activities of daily life.

**Figure 3:**
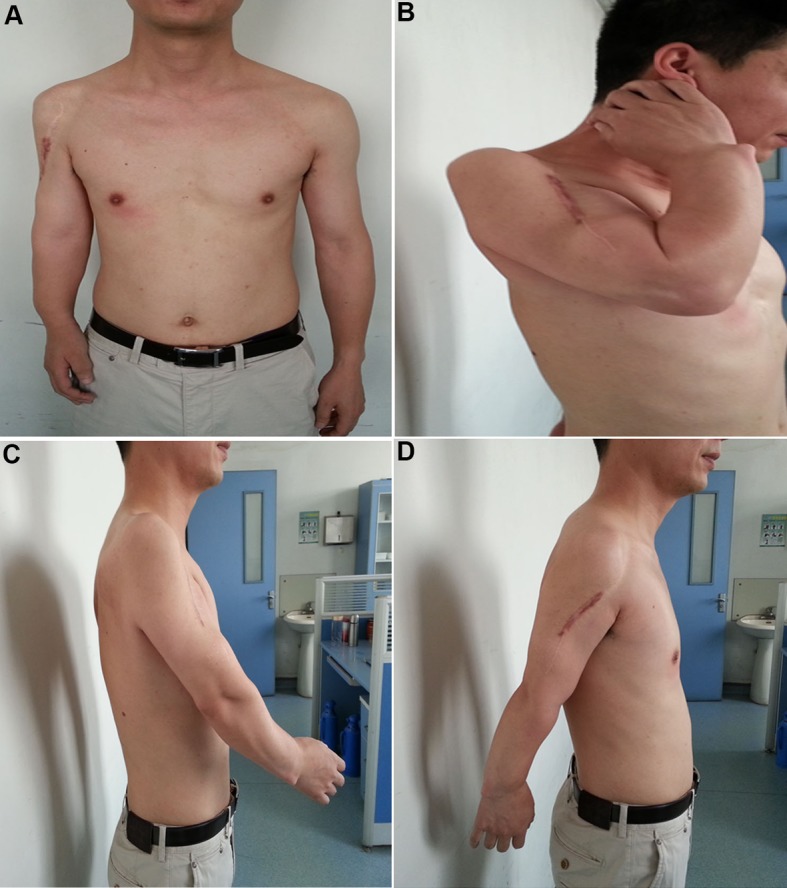
The ranges of motion of the shoulder joint at postoperative 8 years. (A) Overall appearance of the treated arm in contrast to the normal one. (B) Upward rotation at 30° to 40°±. (C) Flexion at 30°-35°±. (D) Extension at 30°-35°±.

During the 8-year follow-up, clinical and radiologic examinations were done at the periodic controls. There are no signs of local recurrence and/or remote metastasis so far. The therapeutic results have been satisfied with a good functional recovery of the treated limb, enabling the patient to return to the pre-disease daily living and occupational activities. The overall functional outcomes were assessed by the musculoskeletal tumor society (MSTS) scoring system. The patient had the overall score 24 in the last examinations of the follow-up, and the ranges of motion of the shoulder joint are shown in Figure 3.

## Discussion

Proximal humerus tumors treated with amputation usually result in a complete loss of the hand/limb function. Limb-salvage procedures have been popularly put into clinical practice in spite of amputations in bone tumor surgery. However, the surgical procedures remain challenges for orthopedic surgeons toward simultaneous replacing the defects of bone/soft tissue and restoring the functions of the involved joints/limbs after tumor resection. Preservation of the functional capacities of the involved upper extremity along with a complete removal of the tumor is considered as the mostly important criteria in the surgical management of the bone tumor of the proximal humerus. Moreover, concerns also should be paid for preservation of length and cosmesis of the upper extremity toward supporting quality of life for the patients. The indications for the prosthetic replacement of the proximal humerus tumors as proposed by Ross et al [15] have been updated, and in cases with no distant metastasis and no direct invasion of the neurovascular bundle, limb-salvage procedures can be performed [16]. Most importantly, the functions of the hand, the elbow, the wrist and the shoulder can be maintained using the limb-salvage surgery with the prosthetic replacement after resection of the tumors. The reconstructive procedures represent an effective surgical strategy for limb salvage in the treatment of large segmental defects following resection of the proximal humerus tumors, substantially solving the functional and esthetic problems due to such a wide resection and significantly improving the quality of life for the patient.

It is worth noting that good function of a treated upper limb with the procedures is largely based on the stability of the shoulder joint, while the stability of the shoulder joint can only be ensured by reliable reconstruction with minimizing the amount of resection of muscle and soft tissue within the complete removal of the tumor during surgery [16]. Various complications can be seen after prosthetic replacement for proximal humeral tumors, for example, an unstable shoulder joint (subluxation or dislocation), local recurrence, loose prostheses, deep infection, nerve injury and more [17]. Nevertheless, none of those complications has happened in this patient so far. In addition, it should be aware that Ollier disease has a high rate of malignant transformation, thus the patients, once identified, require life-long monitoring for clinical symptoms (pain, increase in size, and so on) and radiologic signs of malignant transformation. Early orthopedic treatments in the patients may be beneficial for improvement of overall life quality and life expectancy as a safe and reliable option especially in the patients with primary bone tumors without metastasis, while the patients with metastatic diseases usually have poorer results and short life expectancy. As a result, the stage and progression of tumor, the extent of bone destruction, and the degree of soft tissue invasion along with age and functional condition of the patients must be fully considered when selecting the appropriate surgical technique. All the thoughtful considerations would ensure the rigorous safety with its indications for the surgery.
